# Cellular basis of ClC-2 Cl^−^ channel–related brain and testis pathologies

**DOI:** 10.1074/jbc.RA120.016031

**Published:** 2020-11-23

**Authors:** Corinna Göppner, Audrey H. Soria, Maja B. Hoegg-Beiler, Thomas J. Jentsch

**Affiliations:** 1 Leibniz-Forschungsinstitut für Molekulare Pharmakologie (FMP), Berlin, Germany; 2 Max-Delbrück-Centrum für Molekulare Medizin (MDC), Berlin, Germany; 3 NeuroCure Cluster of Excellence, Charité Universitätsmedizin, Berlin, Germany

**Keywords:** anion channel, infertility, leukoencephalopathy, myelin vacuolization, RPE, HepaCAM, aldosteronism, AMH, anti-Müllerian hormone, APC, adenomatous polyposis coli, CNP, 2,3-cyclic nucleotide phosphodiesterase, GC, germ cell, GFAP, glial fibrillary acidic protein, GLAST, glutamate-aspartate transporter, GlialCAM, glial cell adhesion molecule, H&E, hematoxylin and eosin, HepaCAM, hepatic cell adhesion molecule, IHC, immunohistochemistry, LRRC8, leucine rich repeat containing member 8, MLC, megaencephalic leukoencephalopathy with subcortical cysts, PFA, paraformaldehyde, RPE, retinal pigment epithelium, SC, Sertoli cell, VRAC, volume regulated anion channel

## Abstract

The ClC-2 chloride channel is expressed in the plasma membrane of almost all mammalian cells. Mutations that cause the loss of ClC-2 function lead to retinal and testicular degeneration and leukodystrophy, whereas gain-of-function mutations cause hyperaldosteronism. Leukodystrophy is also observed with a loss of GlialCAM, a cell adhesion molecule that binds to ClC-2 in glia. GlialCAM changes the localization of ClC-2 and opens the channel by altering its gating. We now used cell type–specific deletion of ClC-2 in mice to show that retinal and testicular degeneration depend on a loss of ClC-2 in retinal pigment epithelial cells and Sertoli cells, respectively, whereas leukodystrophy was fully developed only when ClC-2 was disrupted in both astrocytes and oligodendrocytes. The leukodystrophy of *Glialcam*^−/−^ mice could not be rescued by crosses with *Clcn2*^op/op^ mice in which a mutation mimics the “opening” of ClC-2 by GlialCAM. These data indicate that GlialCAM-induced changes in biophysical properties of ClC-2 are irrelevant for *GLIALCAM*-related leukodystrophy. Taken together, our findings suggest that the pathology caused by *Clcn2* disruption results from disturbed extracellular ion homeostasis and identifies the cells involved in this process.

Chloride channels are molecularly very diverse. They can reside in the plasma membrane or intracellular organelles and perform a plethora of functions. These include transepithelial transport, the modulation of cellular excitability, or the regulation of both intracellular and extracellular ion concentrations and of cell volume (1, 2, 3). Accordingly, both loss- and gain-of-function mutations in diverse chloride channel genes, both in humans and animal models, result in a large spectrum of disease phenotypes. These in turn allow conclusions on the physiological roles of individual channels.

ClC-2 (4) is a widely expressed member of the CLC gene family of Cl^−^ channels and 2Cl^−^/H^+^ transporters (2) whose first member, ClC-0, was cloned from electric fish (5). ClC-2 is expressed at the plasma membrane where it mediates inwardly rectifying Cl^−^ currents that are slowly activated by membrane hyperpolarization (4, 6). ClC-2 can also be activated by cell swelling (6), but, in contrast to volume-regulated LRRC8/VRAC anion channels (3, 7, 8), appears to lack a prominent role in cell volume regulation (2), although Bergmann glia from *Clcn2*^−/−^ mice appeared swollen (9). Regions and residues important for the slow opening of ClC-2 by hyperpolarization or cell swelling have been mapped to the amino terminus of ClC-2 (6) and an intracellular loop (10). Mutations in these regions virtually abolish gating and result in large Cl^−^ currents with an almost ohmic behavior. Interestingly, coexpression with the cell adhesion protein GlialCAM (also known as HepaCAM), which can bind ClC-2 and localize it to cell–cell contacts, similarly “opens” ClC-2 by changing its gating (9, 11, 12, 13). However, the physiological role of this effect remains unclear.

To elucidate the physiological roles of ClC-2 we previously generated constitutive ClC-2 KO mice (*Clcn2*^−/−^ mice) (14). These mice display male infertility and blindness due to early postnatal degeneration of the testes and retina, respectively (14). They also slowly develop leukodystrophy (15) in which vacuoles appear in the white matter. These symptoms were reproduced in independently generated *Clcn2*^−/−^ mice (16, 17) and in *Clcn2* mutant mice obtained in a chemical mutagenesis screen (18). Several years later, homozygous *CLCN2* loss-of-function mutations were identified in patients with leukodystrophy, in some cases accompanied by visual problems (19, 20, 21, 22) or azoospermia-related infertility (23). Of note, mutations in *GLIALCAM* cause a related form of leukodystrophy (megalencephalic leukoencephalopathy with subcortical cysts, MLC) (24). MLC can also be caused by mutations in *MLC1*, which encodes a membrane protein of unknown function (25). MLC1, GlialCAM, and ClC-2 apparently form ternary complexes in glial membranes. Loss of either GlialCAM or MLC1 altered localization and decreased ClC-2 expression levels in glia (9), suggesting a loss of ClC-2 function as a common factor in MLC, but it had remained unclear whether the loss of ClC-2 “opening” by GlialCAM contributes to the pathology. To explain the degenerative phenotypes in testis, retina, and brain, we have speculated that ClC-2 regulates the extracellular ion homeostasis in the clefts between cells (9, 14, 15).

Genetic analysis of patients with hyperaldosteronism (26, 27) recently revealed *CLCN2* missense mutations in regions known to affect ClC-2 gating (6, 10). By opening the ClC-2 “gate”, these human mutations drastically increase ClC-2 Cl^−^ currents (27, 28). We generated knockin mice (*Clcn2*^op/op^) in which an N-terminal deletion, based on our previous structure-function analysis (6), opens ClC-2 to a similar degree as aldosteronism-associated human mutations (28). Adrenal zona glomerulosa cells of *Clcn2*^op/op^ mice are strongly depolarized by large Cl^−^ currents, resulting in increased Ca^2+^-influx and stimulation of aldosterone synthesis (28). These *Clcn2*^op/op^ mice can now be used to explore effects of markedly increased Cl^−^ currents in other cells and tissues.

To better understand the mechanisms underlying ClC-2-related disease, we used novel mouse models to genetically identify the cell types that are critically involved in the pathogenesis. Targeted deletion of ClC-2 in Sertoli or retinal pigment epithelial cells revealed that lack of ClC-2 in those cells is responsible for the loss of germ cells and photoreceptors, respectively. Specific *Clcn2* disruption in astrocytes or oligodendrocytes produced no or only mild leukodystrophy, respectively, whereas the full extent of *Clcn2*-related leukodystrophy was reproduced by combined disruption in both cell types. Finally, the leukodystrophy of *Glialcam*^−/−^ mice could not be rescued by crosses with *Clcn2*^op/op^ mice, showing that the lack of opening of ClC-2 by GlialCAM plays no significant role in the pathology of *Glialcam*^−/−^ mice. Our work bolsters the notion that ClC-2 is crucial for extracellular ion homeostasis in various tissues and identifies the cell types responsible for that regulation.

## Results

### Generation of conditional *Clcn2* knockout mice

Conditional *Clcn2*^lox/lox^ mice were generated by homologous recombination in embryonic stem cells. Exons 2 and 3 of the mouse *Clcn2* gene were flanked with loxP sites (Fig. S1, *A* and *B*). Excision of these exons by the Cre recombinase is expected to result in a frameshift and termination of protein synthesis by an early stop codon that occurs before the first transmembrane domain. Western blots of brain lysates revealed that *Clcn2*^lox/lox^ mice expressed normal amounts of ClC-2 protein (Fig. S1*C*). When crossed with *deleter* mice that express the Cre-recombinase in all tissues (29), the brain had lost detectable ClC-2 immunoreactivity just like brain from *Clcn2*^−/−^ mice (Fig. S1*C*). After this validation, *Clcn2*^lox/lox^ mice were used with various cell type–specific Cre lines to identify the cell types causally related to the various degenerative phenotypes of *Clcn2*^−/−^ mice (14).

### Loss of ClC-2 in Sertoli, but not germ cells underlies azoospermia of *Clcn2*^−/−^ mice

Male *Clcn2*^−/−^ mice are infertile owing to early postnatal testicular degeneration and the ensuing azoospermia (14). Infertility associated with azoospermia has also been found in a male patient with subclinical *CLCN2*-related leukodystrophy (23). In *Clcn2*^−/−^ mice, seminiferous tubules fail to develop normal lumina and germ cells do not complete meiosis. Eventually, germ cells of all stages are lost, resulting in a Sertoli cell–only phenotype (14). Based on the immunohistochemical detection of ClC-2 in Sertoli cells, and their abnormal morphology in *Clcn2*^−/−^ mice, we hypothesized that the azoospermia was due to a loss of ClC-2 in Sertoli cells that normally provide vital support to germ cells (14). Considering the nearly ubiquitous expression pattern of ClC-2, other possibilities could not be excluded, however.

**Figure 1:**
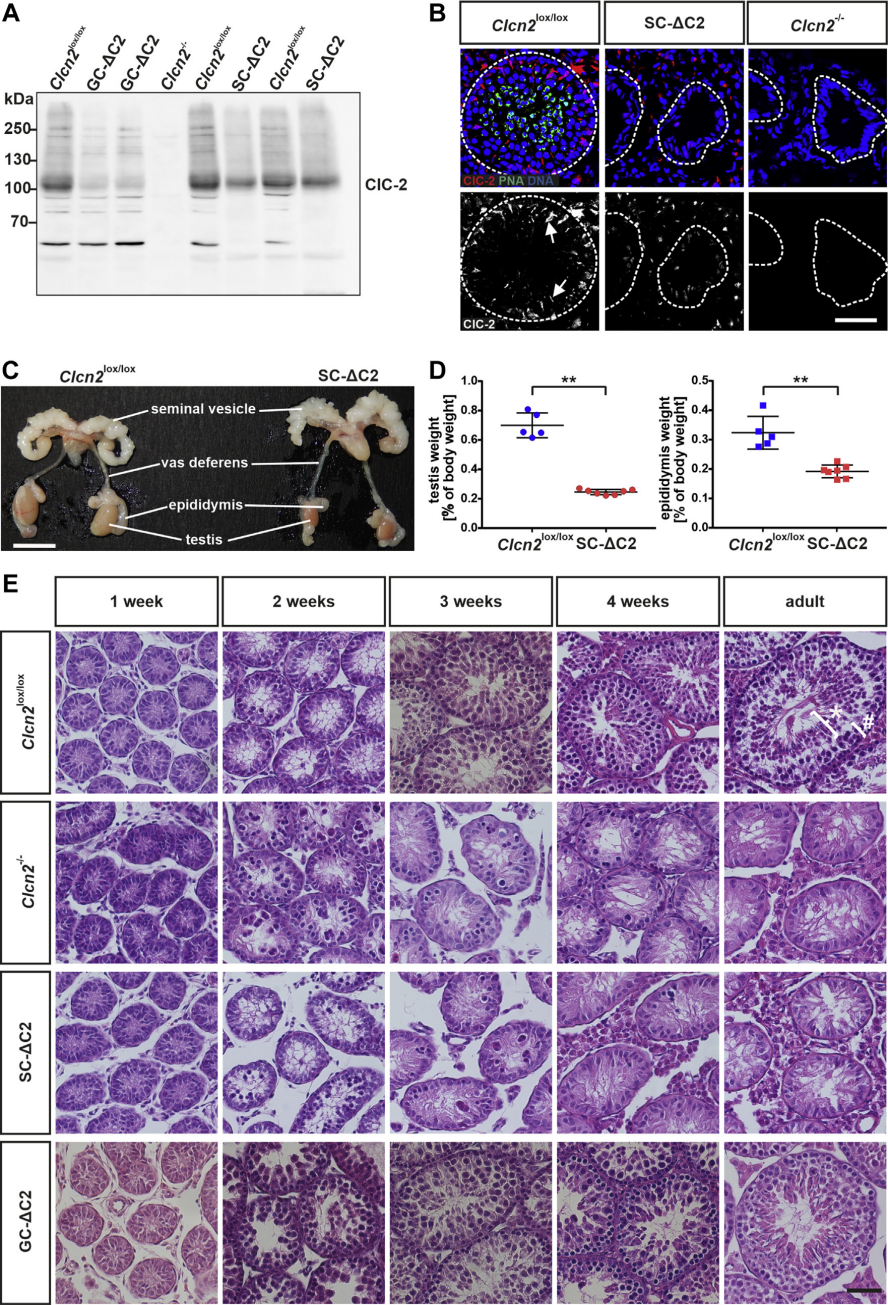
**Sertoli cell–specific *Clcn2* disruption causes testicular degeneration in mice.***A*, Western blot analysis of ClC-2 of membrane fractions isolated from complete testis of adult *Clcn2*^lox/lox^, GC-ΔC2 (germ cell–specific *Clcn2* knockout), *Clcn2*^-/-^ and SC-ΔC2 mice. Equal amounts of protein, as determined by BCA assay, were loaded. This blot is representative for at least two independent experiments. *B*, *upper row*, testis cryosections of adult *Clcn2*^lox/lox^, SC-ΔC2 (Sertoli cell–specific *Clcn2* knockout), and *Clcn2*^-/-^ mice immunolabeled for ClC-2 (*red*) and costained with peanut agglutinin (*green*), a marker for acrosomes of spermatozoa (82). DNA in nuclei was stained with DAPI (*blue*). Dashed lines indicate the outer limit of the seminiferous tubules. *Lower row*, pictures as in upper row, showing ClC-2 staining (*white*). Staining in Sertoli cells (examples) is marked by arrows. The scale bar represents 50 μm. *C*, macroscopic view of the male reproductive system of adult *Clcn2*^lox/lox^ and SC-ΔC2 mice. The scale bar represents 1 cm. *D*, testis and epididymis weights relative to body weight of adult *Clcn2*^lox/lox^ and SC-ΔC2 mice (n = 5–7). Error bars, mean ± S.D. Statistical significance: ∗∗ *p* < 0.01 (Mann-Whitney U test). *E*, H&E-stained paraformaldehyde-fixed testis sections of *Clcn2*^lox/lox^, *Clcn2*^-/-^, SC-ΔC2, and GC-ΔC2 mice of different ages as indicated. n = 1 to 3. The scale bar represents 50 μm. Bracket labeled with ∗ indicates region containing spermatocytes and spermatids, bracket labeled by # spermatogonia and Sertoli cells.

We now crossed *Clcn2*^lox/lox^ mice with AMH-Cre mice (30) to create Sertoli cell–specific *Clcn2* KO mice (named SC-ΔC2 mice), and with Stra8-Cre (31) to generate germ cell–specific GC-ΔC2 KO mice. In Western blot analysis of membrane proteins from whole testes ClC-2 protein levels appeared somewhat more reduced in GC-ΔC2 than in SC-ΔC2 testes (Fig. 1*A*, Fig. S2*A*). The apparent discrepancy to immunofluorescence, which detects ClC-2 in Sertoli, but not germ cells (14) (Fig. 1*B*), might be explained by the easily detectable, patchy expression of ClC-2 in Sertoli cells. Confirming the Sertoli cell–specific deletion of *Clcn2*, these ClC-2-positive patches were no longer visible in SC-ΔC2 testis, whereas ClC-2 could still be detected in cells outside the tubules (Fig. 1*B*).

In adult SC-ΔC2 mice, testes and epididymis were significantly smaller than in the WT (Fig. 1*C*), closely resembling the corresponding phenotype of *Clcn2*^−/−^ mice (14). Accordingly, the weight of both tissues was markedly reduced (Fig. 1*D*). The reduced weight of SC-ΔC2 epididymis might result from the absence of germ cells in adult SC-ΔC2 mice (Fig. 1*E*). The time course of testicular degeneration was examined by hematoxylin and eosin staining (Fig. 1*E*). Similar to *Clcn2*^−/−^ mice, testicular degeneration of SC-ΔC2 mice started at an age of 2 weeks: cells of the tubular lumen were disorganized, with many cells found in the center of the seminiferous tubule rather than being close to its inner walls as in control *Clcn2*^lox/lox^ mice. In 3-week-old SC-ΔC2 mice, clusters of degenerating cells could be observed in some tubules, which were, however, mainly filled with Sertoli cells. Such clusters of degenerating cells had almost disappeared in SC-ΔC2 mice at 4 weeks of age, a time point at which the tubules of control mice were filled with germ cells of different developmental stages. Eventually, adult SC-ΔC2 mice displayed a Sertoli cell–only syndrome. They were unable to produce offspring during their entire lifespan. By contrast, GC-ΔC2 mice showed no signs of testicular degeneration (Fig. 1*E*) and were fertile. Hence, ClC-2 appears to be dispensable in germ cells but is essential for the role of Sertoli cells in maintaining normal spermatogenesis.

### ClC-2 is needed in retinal pigment epithelium to maintain integrity of photoreceptors

*Clcn2*^−/−^ mice are blind because of an early loss of photoreceptors, which is already visible at postnatal day 14 (P14) (14, 18). Although *Clcn2* is expressed both in the retinal pigment epithelium (RPE) and in all layers of the neuronal retina, we speculated that photoreceptors degenerate because of a lack of support from the RPE (14).

**Figure 2:**
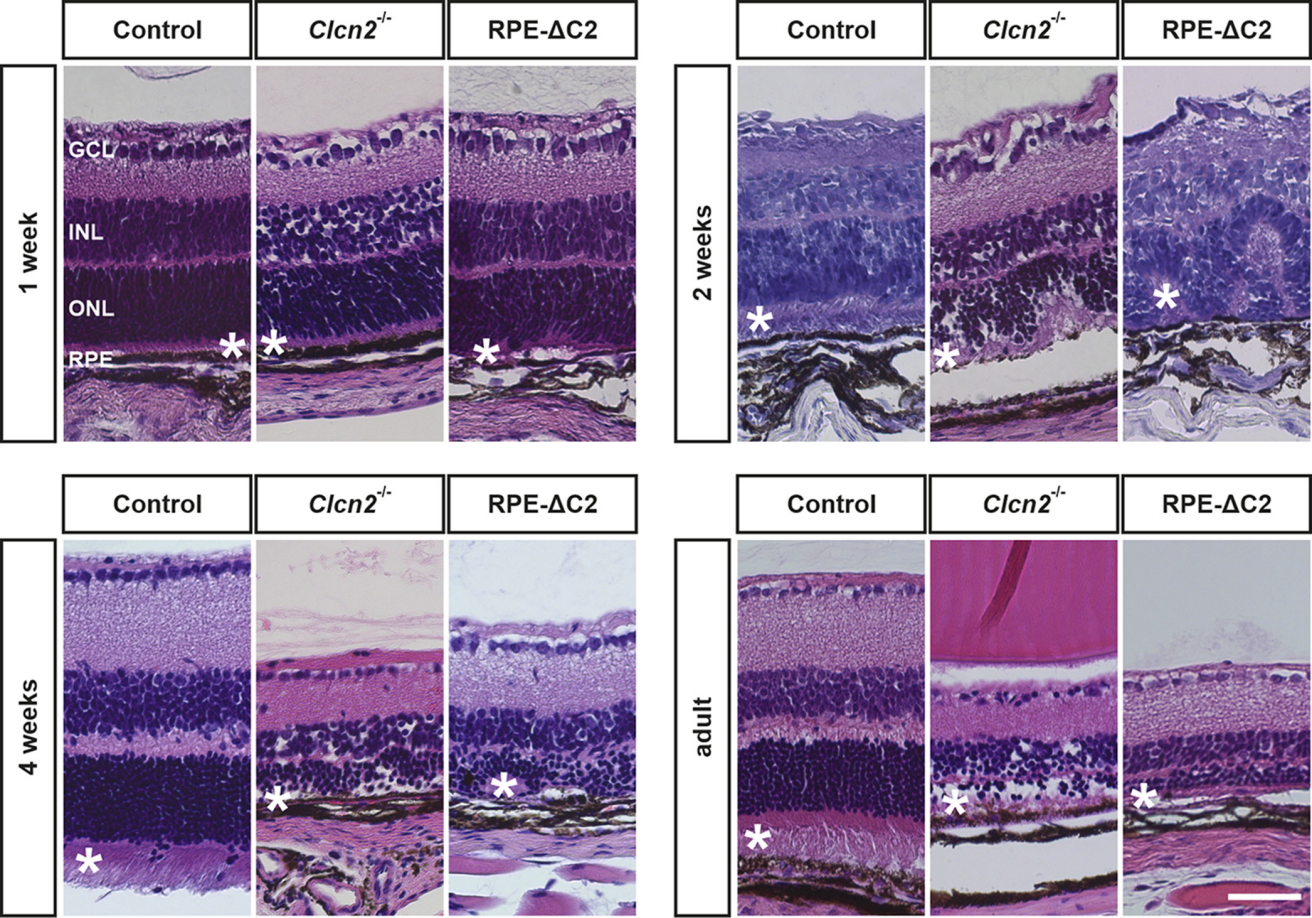
**Retinal degeneration upon *Clcn2* knockout in the retinal pigment epithelium.** H&E-stained retinal sections of 1-, 2-, and 4-week-old and adult control, *Clcn2*^-/-^, and RPE-ΔC2 mice. n = 1 to 3 per age and genotype; ∗ indicates the photoreceptor layer; GCL, ganglion cell layer; INL, inner nuclear layer; ONL, outer nuclear layer; RPE, retinal pigment epithelium. The scale bar represents 50 μm.

To test this hypothesis, we now crossed *Clcn2*^lox/lox^ mice with Trp1-Cre mice (32) to generate RPE-ΔC2 mice that lack ClC-2 specifically in the RPE. These mice displayed retinal degeneration that closely resembled that of *Clcn2*^−/−^ mice (Fig. 2). In 2-week-old mice, the outer nuclear layer and the photoreceptor layer (Fig. 2) appeared disorganized in both RPE-ΔC2 and *Clcn2*^−/−^ mice. At 4 weeks of age, all retinal layers were thinner in either mouse model. However, at this age photoreceptors of *Clcn2*^−/−^ mice were completely lost, whereas residual parts of the photoreceptor layer could still be detected in RPE-ΔC2 mice. This difference might be due to a mosaic-like expression pattern of the Cre-recombinase in Trp1-Cre mice (33), which presumably spared some RPE cells from *Clcn2* ablation. In adult mice, the photoreceptor layer was almost completely lost in either mouse model. These results buttress the hypothesis (14) that the loss of photoreceptors in *Clcn2*^−/−^ mice is secondary to a malfunction of nurturing RPE cells.

### Both oligodendrocytes and astrocytes contribute to leukodystrophy in *Clcn2*^−/−^ mice

Loss of ClC-2 leads to leukodystrophy in both mice (15) and humans (19). In mice, it manifests as spongiform vacuole formation in white matter, which is particularly prominent in the cerebellum (15). ClC-2 is expressed in neurons (34, 35) and glia, where it is found in both astrocytes and oligodendrocytes (9, 15, 36, 37). The presence of vacuoles in myelin sheaths of central neurons of *Clcn2*^−/−^ mice (15) suggests an oligodendrocyte-intrinsic role of ClC-2, but it has remained unclear whether other cell types contribute to *CLCN2*-related leukodystrophy.

**Figure 3:**
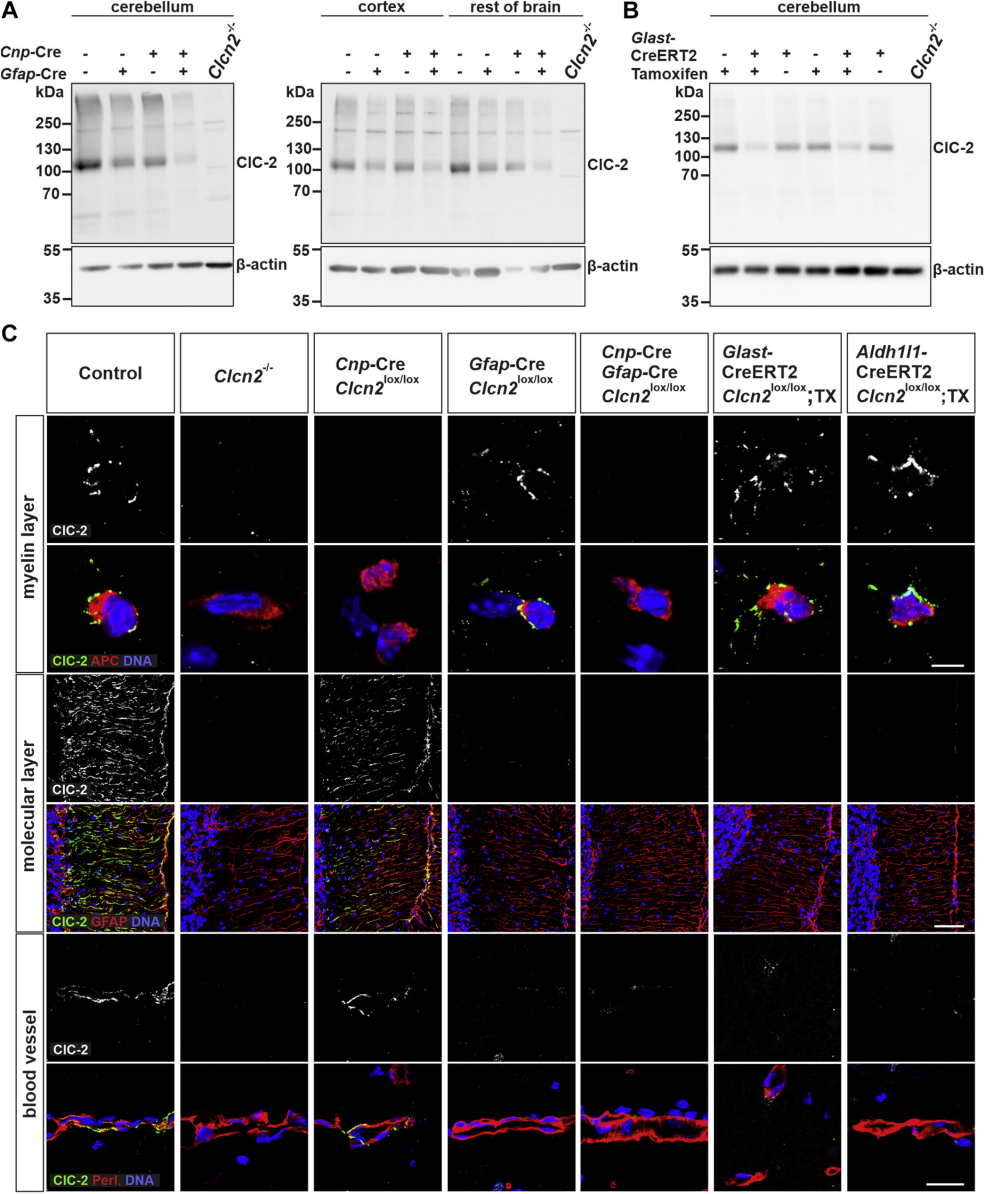
**ClC-2 expression and localization in glial cell–specific *Clcn2* knockout mice.***A* and *B*, Western blot analysis of ClC-2 of membrane fractions isolated from cerebellum, cerebral cortex, and remainder of brain of adult control (*Clcn2*^lox/lox^), oligodendrocyte-specific (Cnp-Cre;*Clcn2*^lox/lox^), astrocyte-specific (GFAP-Cre;*Clcn2*^lox/lox^, GLAST-CreERT2;*Clcn2*^lox/lox^ induced with tamoxifen), oligodendrocyte-/astrocyte-specific (Cnp-Cre;GFAP-Cre;*Clcn2*^lox/lox^) *Clcn2* knockout mice and *Clcn2*^−/−^ mice. Equal amounts of protein, as determined by BCA assay, were loaded with β-actin serving as loading control. This blot is representative for 1 to 3 independent experiments. *C*, immunofluorescent staining of ClC-2 (*green/white*) in brain cryosections of adult control, conditional *Clcn2* knockout as indicated, and *Clcn2*^−/−^ mice. Costaining for adenomatous polyposis coli (*red*) visualizes the cell bodies of oligodendrocytes in the myelin layer of the cerebellum. Costaining for the astrocytic cytoskeletal protein GFAP (*red*) visualizes Bergmann glia on the molecular layer of the cerebellum and costaining for perlecan (*red*) visualizes astrocytic endfeet at blood vessels at the hippocampus region. DNA in the nuclei was stained with DAPI (*blue*). The scale bars represent 5 μm (staining in myelin layer), 50 μm (staining in molecular layer), 20 μm (staining of blood vessels).

**Figure 4:**
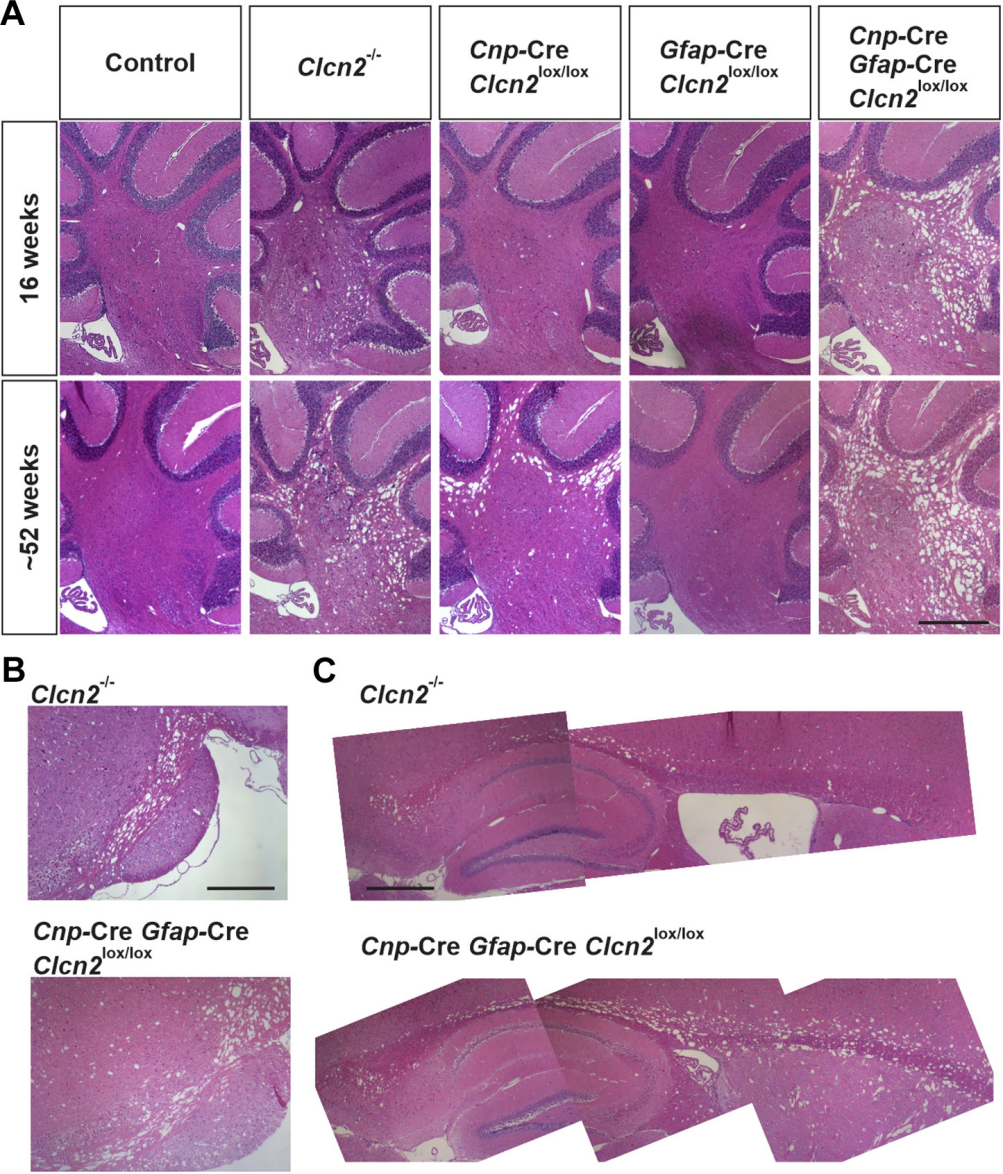
**Myelin vacuolization in glial cell–specific *Clcn2* knockout mouse models.** H&E-stained sagittal brain sections of the myelin layer of the cerebellum of control, *Clcn2*^−/−^, oligodendrocyte-specific (Cnp-Cre;*Clcn2*^lox/lox^), astrocyte-specific (GFAP-Cre;*Clcn2*^lox/lox^), and oligodendrocyte-/astrocyte-specific (Cnp-Cre;GFAP-Cre;*Clcn2*^lox/lox^) *Clcn2* knockout mice in the age of 16 or 52 weeks (*A*), of the brain stem (*B*), and the corpus callosum (*C*) of 52-week-old *Clcn2*^−/−^ and oligodendrocyte-/astrocyte-specific *Clcn2* KO mice. n = 2 to 5. The scale bars represent 500 μm.

To answer this question, we crossed *Clcn2*^lox/lox^ mice to oligodendrocyte-specific Cnp-Cre mice (38) and several Cre lines, including GFAP-Cre mice (39), for deletion in astrocytes. Western blots (Fig. 3, *A* and *B*, Fig. S2*B*) and immunohistochemistry (Fig. 3*C*) ascertained the specificity and efficacy of *Clcn2* disruption. The vacuolization in cerebella of these mice was followed over time in comparison with *Clcn2*^−/−^ and WT mice (Fig. 4*A*). Oligodendrocyte-specific disruption of *Clcn2* was sufficient to generate vacuoles in white matter but failed to fully reproduce the severity of *Clcn2*^−/−^ leukodystrophy. In contrast to *Clcn2*^−/−^ mice, vacuoles in Cnp-Cre;*Clcn2*^lox/lox^ mice were not yet present at 16 weeks of age and were less abundant also in 52-week-old mice. Disruption of *Clcn2* in astrocytes did not cause vacuole formation even at 52 weeks of age, as shown for GFAP-Cre;*Clcn2*^lox/lox^ mice in Fig. 4*A*. Cre-lines targeting astrocytes differ in specificity and efficiency of target deletion, mainly because of the diversity of astrocyte subtypes and precursor cells that are common with neurons. We therefore used two additional, inducible Cre-lines to disrupt *Clcn2* in astrocytes, GLAST-CreERT2 (40) and Aldh1/1-CreERT2 (41). After validating their effect on ClC-2 expression (Fig. 3), we investigated whether *Clcn2*-disruption with these lines (with Cre expression being induced by tamoxifen injection at 3–4 weeks) would result in the appearance of vacuoles in cerebellum. This was not the case, neither at 16 nor 52 weeks of age (Fig. S3). Hence, disruption of *Clcn2* in astrocytes is insufficient to cause overt leukodystrophy.

However, additional disruption in astrocytes significantly worsened the vacuolization seen with oligodendrocyte-specific *Clcn2* disruption. In Cnp-Cre, GFAP-Cre;*Clcn2*^lox/lox^ mice vacuolization in the cerebellum even appeared to be worse than in *Clcn2*^−/−^ mice (Fig. 4*A*). Like in *Clcn2*^−/−^ mice, it extended to other white matter regions of the brain (Fig. 4 *B*–*C* and Fig. S4) and developed with a similar time course (Fig. S5). We conclude that ClC-2 needs to be absent from both oligodendrocytes and astrocytes to reproduce the leukodystrophy of *Clcn2*^−/−^ mice.

### Leukodystrophy caused by *Glialcam* disruption is not due to changes in ClC-2 gating

Loss-of-function mutations not only in *CLCN2* but also in *GLIALCAM* and *MLC1*, with which ClC-2 may form ternary complexes in glia (9, 12), can cause leukodystrophy in humans and mice (9, 24, 25). We have shown previously that disruption of either *Glialcam* or *Mlc1* led to mistargeting and decreased abundance of ClC-2 in glia (9). As GlialCAM increases Cl^−^ current amplitudes by virtually abolishing ClC-2 gating, we expected a change from linear to inwardly rectifying, smaller ClC-2 currents in *Glialcam*^−/−^ mice. This was indeed observed in oligodendrocytes, but surprisingly not in Bergmann glia (9).

We now asked whether the effect of GlialCAM on ClC-2 gating plays a significant role in *GLIALCAM*-related leukodystrophy. We crossed *Glialcam*^−/−^ with *Clcn2*^op/op^ mice (28), which express an N-terminal deletion mutant (6) that opens ClC-2 to roughly the same degree as the binding to GlialCAM (11, 12, 28). We argued that a cross with *Clcn2*^op/op^ mice may rescue the leukodystrophy of *Glialcam*^−/−^ mice if it is predominantly caused by a lack of ClC-2 opening.

GlialCAM interacts with ClC-2 through its extracellular amino terminus and the first part of its single transmembrane span rather than through its cytosolic C terminus (12, 42). Accordingly, GlialCAM directs ΔN-ClC-2, a mutant carrying a deletion in the cytosolic N terminus similar to that of ClC-2^op^, to cell–cell contacts just like WT ClC-2 (12). Also *in vivo*, no differences in the subcellular localization of WT and ClC-2^op^ were found (Fig. S6*A*). Like WT ClC-2 (9, 15, 36), the mutant ClC-2^op^ protein was prominently detected on cell bodies of cerebellar oligodendrocytes, in Bergmann glia, and at astrocytic endfeet that contact blood vessels. Agreeing with previous results for WT ClC-2 (9), Western blots of cerebellum revealed a marked reduction of both WT and mutant ClC-2^op^ protein in the absence of GlialCAM (Fig. S6*B*), whereas no change was seen in the remainder of the brain (Fig. S6*C*). Hence, as described for other tissues (28), the N-terminal deletion affected neither the abundance nor localization of ClC-2.

**Figure 5:**
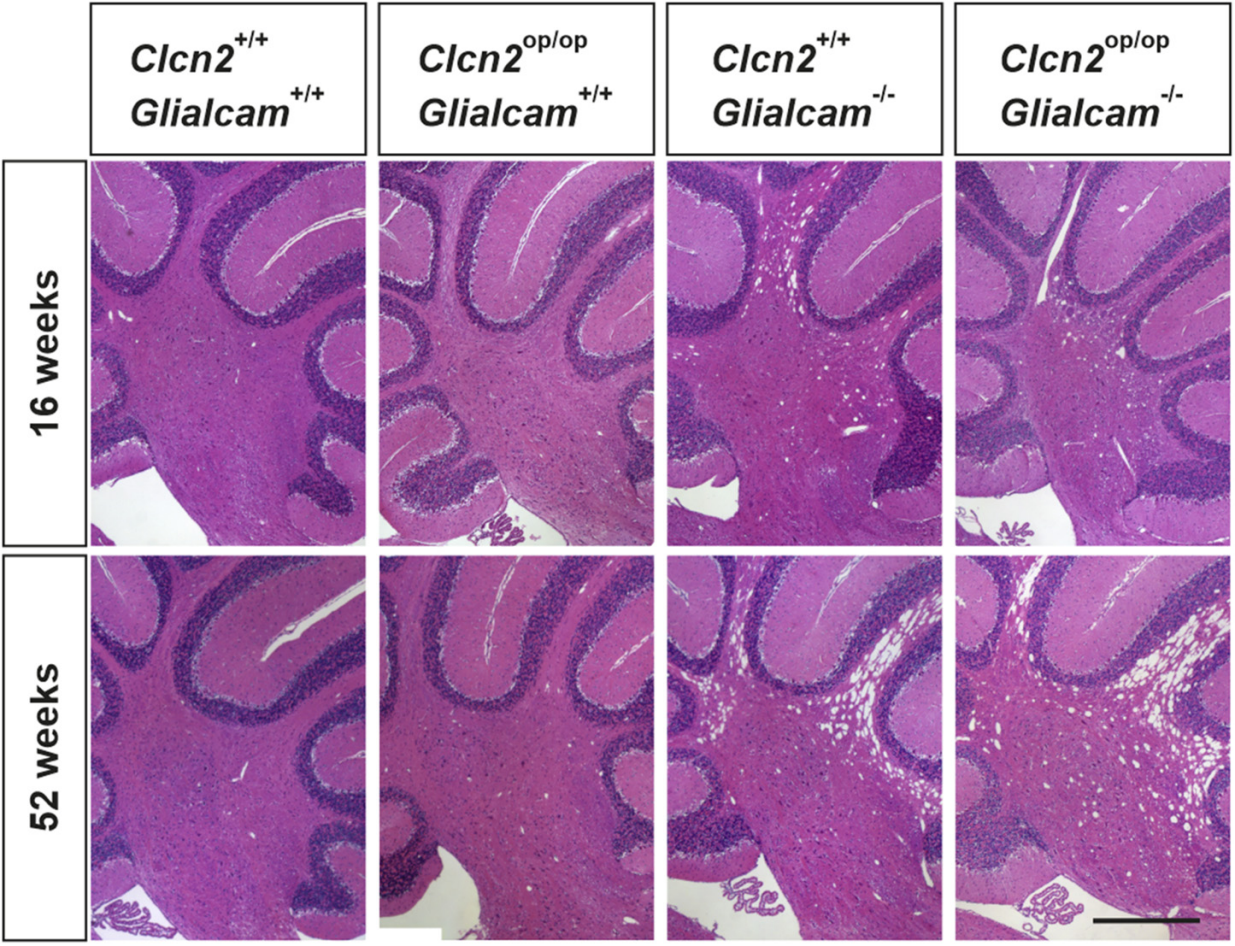
**Similar myelin vacuolization phenotype of *Clcn2***^**op/op**^***Glialcam***^**−/−**^**compared with *Glialcam***^**−/−**^**mice.** H&E-stained paraformaldehyde-fixed sagittal brain sections (myelin part of the cerebellum) of wildtype (*Clcn2*^+/+^;*Glialcam*^+/+^), *Clcn2*^op/op^;*Glialcam*^+/+^, *Clcn2*^+/+^;*Glialcam*^−/−^, and *Clcn2*^op/op^;*Glialcam*^−/−^ mice at the age of 16 and 52 weeks. n = 1 to 3. The scale bar represents 500 μm.

The morphology of *Clcn2*^op/op^ brain, including the cerebellum, appeared normal (Fig. 5). As described previously (9), cerebella from *Glialcam*^−/−^ mice developed vacuolization that was visible at 16 weeks and progressed further with age. The extent and progression of vacuolization was indistinguishable in *Clcn2*^op/op^;*Glialcam*^−/−^ brain (Fig. 5), refuting the hypothesis that *GLIALCAM*-related leukodystrophy is due to the lack of GlialCAM-induced opening of ClC-2.

## Discussion

Although ClC-2 is nearly ubiquitously expressed across mammalian tissues and cells, loss of this Cl^−^ channel causes only a limited number of significant pathologies in mice and men. All of these are related to cellular degeneration. In two cases, cellular degeneration is not a cell-autonomous consequence of *Clcn2* disruption but results from ClC-2 deletion in closely apposed neighboring cells on which the degenerating cells depend for their function and survival: loss of ClC-2 in Sertoli cells leads to the degeneration and eventual loss of male germ cells, whereas *Clcn2* disruption in retinal pigment epithelial cells entails a rapid loss of photoreceptors. With *CLCN2*-related leukodystrophy the situation is more complex: *Clcn2* disruption in oligodendrocytes led cell-autonomously to myelin vacuolization, whereas additional deletion in astrocytes, which form gap junction–connected syncytia with oligodendrocytes (43, 44), was required to achieve the same extent of vacuolization observed with global *Clcn2* disruption. Our data suggest that all three pathologies arise from defective regulation of the *milieu extérieur* in narrow clefts between cells in the absence of ClC-2.

### Degeneration of the testis and the retina

Both testes and retina are highly differentiated tissues in which epithelial cells provide crucial support to extraordinarily specialized cells, germ cells and photoreceptors, respectively. In testis, Sertoli cells control the ionic composition of the tubular lumen and the fluid surrounding germ cells during their maturation; provide growth factors and lactate, the central energy metabolite for germ cells (45, 46, 47); and phagocytose residual bodies of spermatids that shed a large portion of their cytoplasm on their way to fully differentiated spermatozoa (48, 49). Tight junctions between Sertoli cells form the blood–testis barrier and create an immune-privileged compartment (50). Similarly, retinal pigment epithelial (RPE) cells are connected by tight junctions and form the outer retina–blood barrier. Transepithelial solute transport by the RPE controls the composition of the fluid surrounding photoreceptors and is critical for their survival (51). RPE cells buffer ions released from photoreceptors during electrical activity, provide glucose to photoreceptors while removing its metabolite lactate, participate in the visual cycle that regenerates the visual pigment retinal, phagocytose outer segments from photoreceptors, and secrete growth factors (51, 52). Not surprisingly, impairment of RPE function can lead to several blinding diseases (52).

In *Clcn2*^−/−^ mice, degeneration of germ cells and photoreceptors start around the time when Sertoli or RPE cells establish the respective blood–organ barrier (14). This observation had led us to hypothesize that the loss of ClC-2 in these cells, rather than in germ cells or photoreceptors, triggers the observed degeneration of cells beyond that barrier (14). This idea was further supported by abnormal morphology of Sertoli cells in adult *Clcn2*^−/−^ mice and by a strong reduction of transepithelial current, voltage, and electrical resistance across their retinal pigment epithelium (14) at P36, with the caveat that the retina has already partially degenerated at this age. Abnormal RPE function is also indicated by longer apical RPE microvilli in chemically induced *Clcn2*^−/−^ mice before retinal degeneration at P10 and by electrooculograms of heterozygous mice (18). These recordings revealed changed light peaks thought to originate in the RPE. However, these observations cannot prove that Sertoli or RPE cells are at the origin of the degeneration of germ and photoreceptor cells, which both express *Clcn2* (14), with germ cells actually expressing somewhat more ClC-2 protein than Sertoli cells (Fig. 1*A*). Using cell type–specific disruption of *Clcn2* we now show that the lack of ClC-2 in Sertoli cells, but not germ cells, recapitulates the testicular phenotype of *Clcn2*^−/−^ mice and that *Clcn2* disruption in RPE cells is sufficient to explain their retinal degeneration.

Our results demonstrate that the degeneration observed in testis and retina is owed to the lack of ClC-2 in “nursing” Sertoli and RPE cells. These cells provide the correct environment for adjoining germ cells and photoreceptors, respectively, which reside behind a blood–organ barrier. Further similarities are the phagocytic activity of Sertoli and RPE cells (53), which remove residual bodies from spermatids and outer segment of photoreceptors, respectively, and their involvement in lactate transport. Lactate is generated by and released from Sertoli cells as nutrient for germ cells, whereas it needs to be removed from photoreceptors where it arises as a waste metabolite. Lactate crosses membranes through H^+^-lactate MCT (SLC16) cotransporters (54). H^+^-cotransport is a burden for the pH regulation of the cytosol and particularly of the much smaller extracellular clefts in these tissues. Extracellular acidification upon coupled H^+^-lactate exit might be compensated by Cl^−^/HCO_3_^−^ exchange that requires Cl^−^ recycling across the membrane (15). ClC-2, which is activated by acidic extracellular pH (6) and high intracellular Cl^−^ concentration (55, 56, 57), would be ideally suited for this task. In retina, it might also play an indirect role in buffering extracellular cations during electrical activity, akin to its postulated role in glia (15).

Two other anion channels were implicated in male infertility or retinal degeneration. Ablation of the volume-regulated anion channel VRAC (7) in germ cells, but not in Sertoli cells, causes male infertility in mice (58). As both ClC-2 and LRRC8/VRAC channels are expressed in either cell type, the dependence of degeneration on different cell types points to disparities in channel properties or regulation. Unlike ClC-2, VRAC is almost closed at rest, insignificantly affected by pH_o_, strongly activated by cell swelling, and modulated by several signaling cascades (3, 59, 60, 61, 62). Unlike ClC-2, VRAC also conducts organic compounds including lactate, amino acids, and signaling molecules (59, 60, 62, 63). Disruption of *Clcn2* causes azoospermia, whereas loss of *Lrrc8a* results in abnormal, immotile spermatozoa (58, 64). Consistent with impaired cell volume regulation, late-stage *Lrrc8a*^−/−^ spermatids were swollen (58). In retina, loss of bestrophin1, an anion channel activated by Ca^2+^ or ATP (65) that resides in basolateral membranes of RPE cells, leads to macular dystrophy (66). Its disruption in patients, but strangely not in mice (67), decreases a light peak in electrooculograms that is attributed to the RPE (68) and that is also changed in heterozygous chemically induced *Clcn2*^+/−^ mice (18). This similarity might suggest that both Best-1 and ClC-2 reside in the same membrane. Unfortunately, we ignore the polarization of ClC-2 expression in the RPE. Its basolateral localization in colon (28, 57, 69, 70) might suggest an apical expression in the RPE as many normally basolateral proteins localize to the apical membrane of these cells (51).

### ClC-2 in glia and leukodystrophy

ClC-2 is found both in neurons and glia (36) as ascertained by knockout controlled immunohistochemistry (9, 15). ClC-2 is prominently expressed in Bergmann glia and in myelin of the cerebellar medulla (15). ClC-2 is also present at astrocytic endfeet contacting blood vessels and at the circumference of oligodendrocytic somata where it partially colocalizes in puncta with the gap junction protein Cx47 (9, 15). Of note, astrocytic endfeet also express the water channel AQP4 and the K^+^-channel Kir4.1, which is also expressed on somata of oligodendrocytes (9, 71, 72, 73, 74). Together with connexins, these transport proteins may have a role in buffering extracellular potassium in brain (“K^+^ siphoning”). In this model (75), K^+^ ions that leave neurons during the repolarization of action potentials must be removed quickly from the small clefts between cells. Otherwise, extracellular K concentration ([K^+^]_o_) could rise to levels that depolarize neurons sufficiently to elicit action potentials. After being taken up by glia, e.g., by Kir4.1, K^+^ is buffered in oligodendrocytes and astrocytes that are connected by gap junctions to form a syncytium (43, 44). Kir4.1 and AQP4 finally equilibrate potassium and water with the blood at astrocytic endfeet. This model is supported by the leukodystrophy that arises in mice upon ablation of Kir4.1 (72, 76) or of both Cx32 and Cx47 (75, 77). Crosses between these mouse models revealed that these proteins operate in the same pathophysiological pathway (75).

These similarities in cellular localization and phenotypes led us to hypothesize that the myelin vacuolization of *Clcn2*^−/−^ mice is likewise owed to impaired K^+^ siphoning (15). ClC-2 might provide a neutralizing current for bulk, *per se* electrogenic, channel-mediated K^+^ transport into and out of glia. Since cytoplasmic Cl^−^ concentrations of astrocytes are generally above electrochemical equilibrium (78), Cl^−^ efflux could accompany K^+^ efflux at astrocytic endfeet. Cl^−^ influx into astrocytes through ClC-2 seems only feasible if they are sufficiently depolarized by an increase of [K^+^]_o_ during neuronal activity. A depolarization would, however, lower the open probability of ClC-2 unless it were “opened” by GlialCAM. ClC-2-mediated Cl^−^-influx into oligodendrocytes is less problematic since their intracellular Cl^−^ concentrations are close to equilibrium at normal [K^+^]_o_ (78) and because GlialCAM is known to linearize ClC-2 currents in these cells (9).

Compatible with our hypothesis, glia-restricted disruption of ClC-2 was sufficient to recapitulate the spongiform myelin vacuolization of *Clcn2*^−/−^ mice. Loss of ClC-2 in oligodendrocytes, which form the myelin sheaths in which vacuoles appear (9, 15), sufficed to cause vacuolization, albeit not to the same extent as the global lack of ClC-2. This required additional *Clcn2* disruption in astrocytes, which *per se* did not provoke visible abnormalities as ascertained with three different astrocyte-targeting Cre lines. Hence, *Clcn2* needs to be disrupted in most or all cells of the oligodendrocyte–astrocyte syncytium to yield the full-blown leukodystrophy of *Clcn2*^−/−^ mice. The role of this syncytium is also evident with the leukoencephalopathy observed with disruption of both Cx32 and Cx47 (75, 77), which connect the cells of the syncytium, or of Kir4.1, which appears to have a role in K^+^ siphoning both in oligodendrocytes (74) and astrocytes (79). Somewhat surprisingly, in Cnp-Cre, GFAP-Cre;*Clcn2*^lox/lox^ mice myelin vacuolization appeared earlier and was more pronounced than in *Clcn2*^−/−^ mice. Although we cannot exclude effects of the genetic background, this observation points to a role of ClC-2 in neurons, the main cell type of Cnp-Cre;GFAP-Cre;*Clcn2*^lox/lox^ brain in which ClC-2 is not deleted. Interestingly, and in accord with the K^+^-siphoning model, myelin vacuolization in Cx32/Cx47 double KO mice depends strongly on neuronal activity (75). Hence, our data are compatible with lower overall neuronal activity in *Clcn2*^−/−^ mice, a notion that contradicts the previous beliefs that loss of ClC-2 function causes epilepsy, but is consistent with the hyperexcitability of inhibitory interneurons in *Clcn2*^−/−^ mice (35).

Using conditional *Clcn2* KO mice, our work has established the cell types in which ClC-2 disruption causes the KO pathology. One may ask whether other cell types participate in the KO phenotype. This is obviously the case, as for instance, germ cells, in which ClC-2 appears dispensable, degenerate upon loss of ClC-2 in Sertoli cells, or because myelin vacuolization depends on neurons without which myelin sheaths are not formed (in addition to the surprising mitigating effect of neuronal *Clcn2* disruption discussed above). Phenotypes depend on the complex interplay of cells within organisms. Conditional gene KO allows one to pinpoint those cells in which the lack of a specific gene precipitates pathology at the organismal level.

A peculiarity of glial ClC-2 is its coexpression with GlialCAM, which tethers, by homophilic interactions of GlialCAM expressed on either cell, the channel to cell–cell contacts (9, 12). GlialCAM also increases ClC-2 current amplitudes and nearly abolishes its rectification (11, 12). Since *GLIALCAM* mutations entail a vacuolating human leukoencephalopathy (24), we previously generated *Glialcam*^−/−^ mice (9) to study its interaction with ClC-2 and with MLC1, another binding partner of GlialCAM (24) that also underlies human leukoencephalopathy (80). All three mouse models (*Clcn2*^−/−^, *Glialcam*^−/−^, and *Mlc1*^−/−^ mice) displayed myelin vacuolation (9). Disruption of either *Glialcam* or *Mlc1* led to a striking mislocalization of ClC-2 in Bergmann glia and to a strong reduction of ClC-2 protein levels in the cerebellum but not in the rest of the brain. ClC-2 Cl^−^ currents in oligodendrocytes *in situ* were not only reduced in amplitude but displayed the expected change from an almost linear, ClC-2/GlialCAM-mediated current in WT mice to the typical hyperpolarization-activated ClC-2 current that is observed without Glialcam (9). Rather mysteriously, however, these changes in ClC-2 currents were not seen in Bergmann glia that normally expresses both ClC-2 and GlialCAM. Overall, we concluded that changes in ClC-2 properties and/or abundance contribute to the myelin pathology of *Glialcam*^−/−^ or *Mlc1*^−/−^ mice but that GlialCAM and Mlc1 proteins have additional effects as well (9).

We now asked specifically whether the GlialCAM-induced change in biophysical characteristics of ClC-2 has a role in *Glialcam*^−/−^ leukodystrophy. Crossing *Clcn2*^op/op^ mice that express a mutant, open ClC-2 channel (28) with *Glialcam*^−/−^ mice should restore the open phenotype to oligodendrocyte-expressed ClC-2 and open ClC-2 also in cells like Bergmann glia in which no effect of GlialCAM on ClC-2 gating could be observed (9). The answer is clear: opening ClC-2 by mutagenesis does not rescue the leukodystrophy caused by the loss of GlialCAM. We conclude that it is rather the mislocalization or the reduction of ClC-2 protein expression that extends to the ClC-2^op^ mutant (Fig. S6), which is responsible for GlialCAM-related leukodystrophy, together with the loss of other currently unknown functions of GlialCAM. Indeed, the more severe myelin vacuolization of *Glialcam*^−/−^;*Clcn2*^−/−^ compared with *Glialcam*^−/−^ or *Clcn2*^−/−^ mice suggests that impaired or lost ClC-2 function is not the only factor in *Glialcam*-related pathology (9).

## Conclusions

Using cell type–specific disruption of *Clcn2* in mice, we identified those cell types in which ClC-2 must be present in order to prevent the degenerative pathologies observed upon the loss of the channel, i.e., azoospermia, photoreceptor degeneration, and spongiform myelin vacuolization. Given the similarities in mouse and human pathologies, our findings most likely can be extrapolated directly to human patients with loss-of-function mutations in *CLCN2*. The loss of germ cells and photoreceptors is not owed to a cell-autonomous loss of the channel in these cells, but rather to the loss in “nurturing” Sertoli and RPE cells. In glia, loss of ClC-2 in oligodendrocytes cell-autonomously leads to vacuole formation in myelin, but there is an important contribution also of astrocytes with which oligodendrocytes form a large syncytium that provides the right environment for neurons. Perhaps not surprisingly, a report on a different *Clcn2*^−/−^ mouse model also found signs of modest neuronal degeneration (16), which was not found in our studies (9, 15).

We propose as common denominator for the *Clcn2*-related testicular, retinal, and glial pathologies a role of ClC-2 in the regulation of the *milieu extérieur*, which can be altered already by small changes in ion transport rates if the relevant volume is small. Indeed, in many cases, and certainly here, the fluid volume in extracellular clefts is much smaller than that of the cytoplasm. Unfortunately, no reliable methods to quantitatively measure ion concentrations in these narrow clefts are currently available. Although the widely expressed ClC-2 has other important roles, such as transepithelial transport in the intestine (70, 81) and—with pathological gain-of-function mutations—in aldosterone production (26, 27, 28), it appears to be crucial in extracellular ion homeostasis in brain and testis, tissues showing severe degenerative changes upon its ablation in mice and men.

## Experimental procedures

### Mice

All animal experiments were approved and in compliance with the Berlin authorities (LAGeSo). All mice were housed under standard conditions in the animal facility of the MDC according to institutional guidelines. They had access to food and water *ad libitum*.

To generate *Clcn2*^lox/lox^ mice, a 10.6-kb fragment of R1-ES cell genomic DNA containing exons 1 to 22 of *Clcn2* was cloned into pKO Scrambler Plasmid 901 (Lexicon Genetics Inc). A neomycin-resistance cassette flanked by FRT sites was inserted between exons 1 and 2. In a previously generated *Clcn2*^lox/lox^ mouse model introduction of a neomycin cassette at this site led to missplicing and hypomorph expression of the *Clcn2* mRNA. To prevent this, we mutated a putative splice acceptor site downstream of the neomycin cassette (gt→ca). Two loxP sites were inserted flanking exons 2 and 3. To aid Southern-blot detection of the targeted allele an additional EcoRV-site was introduced adjacent to the second loxP site. The linearized vector was electroporated into R1 ES cells and neomycin-resistant clones were screened by Southern blotting. Correctly targeted clones were injected into C57BL/6 blastocysts by the MDC transgenic facility, and the resulting chimeric animals were crossed to *FLP*-deleter mice (in C57BL/6 background) to remove the neomycin-resistance cassette. For PCR genotyping primers P1 together with P2 or P3 with P4 were used to detect the loxP sites.

The generation and validation of the following mouse models have been described as indicated: *Clcn2*^−/−^ mice (14); *Clcn2*^op/op^ mice (28); *Glialcam*^−/−^ mice (9); AMH-Cre mice (30); Stra8-Cre (31); Trp1-Cre (32); Cnp-cre (38); GFAP-Cre (39); GLAST-CreERT2 (40); Aldh1/1-CreERT2 (41). To induce the activity of GLAST-CreERT2 and Aldh1/1-CreERT2, tamoxifen was used: tamoxifen (SIGMA, T-5648) was dissolved in corn oil (SIGMA, C-8267) to prepare a final concentration of 10 mg/ml. Mice in the age of 3 to 4 weeks were intraperitoneally injected daily with 100 mg tamoxifen per kilogram body weight for five consecutive days.

### Antibodies

The rabbit anti-mouse ClC-2 antibodies against C-terminal peptides of mouse ClC-2 (1:500 (HGLPREGTPSDSDDKSQ) for immunohistochemistry [IHC] and 1:1000 (WGPRSRHGLPREGTPSDSDDKSQ) for WB) have been described previously and have been controlled using *Clcn2*^−/−^ tissues (9). The following commercial antibodies have been used: mouse anti-APC (IHC; 1:200, OP80, Millipore), mouse anti-GFAP (IHC, 1:500, G3893, Sigma) rat anti-Perlecan (IHC, 1:2000, MAB1948P, Millipore), mouse anti-β-actin (WB, 1:10,000, A2228, Sigma) and peanut agglutinin conjugated to Alexa Fluor 488 (IHC, 1:1000, Molecular Probes). Labeled secondary antibodies were Alexa Fluor 488 goat anti-rabbit (A11034, Invitrogen), Alexa Fluor 555 goat anti-rabbit (A21429, Invitrogen), Alexa Fluor 555 goat anti-mouse (A21424, Invitrogen).

### Immunohistochemistry

Immunohistochemical staining was performed on cryosections from organs of perfused (1% paraformaldehyde [PFA]/PBS) mice. The air-dried sections were fixed with 1% PFA/PBS for 10 min. The fixation step was stopped using 30 mM glycine. After washing with PBS, the sections were permeabilized with 0.2% Triton-X100/PBS. After blocking with 3% BSA, 0.1% Tween 20 in PBS for at least 30 min at RT, the sections were incubated with primary antibody in blocking buffer overnight at 4 °C in a humidified chamber. After washing with PBS, the sections were incubated in the dark for 1 h at RT with secondary antibody solution (1:1000 in blocking buffer) and DAPI. Confocal images were taken using a Zeiss LSM 510 META or Zeiss LSM 880 laser scanning microscope and the ZEN software (Zeiss). Image processing was done using ZEN software (Zeiss).

### Histology

For histological analysis, mice were perfused with 1% or 4% PFA/PBS under deep anesthesia and the organs were postfixed with 1% or 4% PFA/PBS overnight at 4 °C. On 6- to 7-μm sections of the paraffin-embedded organs, hematoxylin-eosin staining was performed. Images were taken with an AxioCam MRc5 (Zeiss) on an Axiophat microscope (Zeiss) with an ACHROSTIGMAT 5x/0.12, Plan-NEOFLUAR 10x/0.30, or Plan-NEOFLUAR 40x/0.75 objective using the ZEN software (Zeiss). Image processing was done using Image Composite Editor (Microsoft) and ZEN software (Zeiss).

### Western blot analyses

Membrane fractions were isolated from frozen mouse tissue as described previously (9). Protein-containing pellets were resuspended in 50 mM Tris pH 6.8, 140 mM NaCl, 0.5 mM EDTA, and 1% SDS and 1% Triton-X100 with protease inhibitors (4 mM Pefabloc and Complete EDTA-free protease inhibitor cocktail, Roche) by sonification. The protein content was determined using bicinchinonic acid assay, and equal amounts of protein were separated by SDS-PAGE and blotted onto nitrocellulose. Image Studio Lite Ver 5.2 was used to quantify ClC-2 protein bands.

## Data availability

The relevant data are contained within the manuscript.

## Conflict of interest

The authors declare that they have no conflicts of interest with the contents of this article.

## References

[1] Jentsch T.J., Stein V., Weinreich F., Zdebik A.A. (2002). Molecular structure and physiological function of chloride channels. Physiol. Rev..

[2] Jentsch T.J., Pusch M. (2018). CLC chloride channels and transporters: structure, function, physiology, and disease. Physiol. Rev..

[3] Jentsch T.J. (2016). VRACs and other ion channels and transporters in the regulation of cell volume and beyond. Nat. Rev. Mol. Cell Biol..

[4] Thiemann A., Gründer S., Pusch M., Jentsch T.J. (1992). A chloride channel widely expressed in epithelial and non-epithelial cells. Nature.

[5] Jentsch T.J., Steinmeyer K., Schwarz G. (1990). Primary structure of *Torpedo marmorata* chloride channel isolated by expression cloning in *Xenopus* oocytes. Nature.

[6] Gründer S., Thiemann A., Pusch M., Jentsch T.J. (1992). Regions involved in the opening of CIC-2 chloride channel by voltage and cell volume. Nature.

[7] Voss F.K., Ullrich F., Münch J., Lazarow K., Lutter D., Mah N., Andrade-Navarro M.A., von Kries J.P., Stauber T., Jentsch T.J. (2014). Identification of LRRC8 heteromers as an essential component of the volume-regulated anion channel VRAC. Science.

[8] Qiu Z., Dubin A.E., Mathur J., Tu B., Reddy K., Miraglia L.J., Reinhardt J., Orth A.P., Patapoutian A. (2014). SWELL1, a plasma membrane protein, is an essential component of volume-regulated anion channel. Cell.

[9] Hoegg-Beiler M.B., Sirisi S., Orozco I.J., Ferrer I., Hohensee S., Auberson M., Gödde K., Vilches C., de Heredia M.L., Nunes V., Estévez R., Jentsch T.J. (2014). Disrupting MLC1 and GlialCAM and ClC-2 interactions in leukodystrophy entails glial chloride channel dysfunction. Nat. Commun..

[10] Jordt S.E., Jentsch T.J. (1997). Molecular dissection of gating in the ClC-2 chloride channel. EMBO J..

[11] Jeworutzki E., Lagostena L., Elorza-Vidal X., López-Hernández T., Estévez R., Pusch M. (2014). GlialCAM, a CLC-2 Cl^−^ channel subunit, activates the slow gate of CLC chloride channels. Biophys. J..

[12] Jeworutzki E., López-Hernández T., Capdevila-Nortes X., Sirisi S., Bengtsson L., Montolio M., Zifarelli G., Arnedo T., Müller C.S., Schulte U., Nunes V., Martínez A., Jentsch T.J., Gasull X., Pusch M. (2012). GlialCAM, a protein defective in a leukodystrophy, serves as a ClC-2 Cl^−^ channel auxiliary subunit. Neuron.

[13] Maduke M.C., Reimer R.J. (2012). Biochemistry to the rescue: a ClC-2 auxiliary subunit provides a tangible link to leukodystrophy. Neuron.

[14] Bösl M.R., Stein V., Hübner C., Zdebik A.A., Jordt S.E., Mukhophadhyay A.K., Davidoff M.S., Holstein A.F., Jentsch T.J. (2001). Male germ cells and photoreceptors, both depending on close cell-cell interactions, degenerate upon ClC-2 Cl^−^-channel disruption. EMBO J..

[15] Blanz J., Schweizer M., Auberson M., Maier H., Muenscher A., Hübner C.A., Jentsch T.J. (2007). Leukoencephalopathy upon disruption of the chloride channel ClC-2. J. Neurosci..

[16] Cortez M.A., Li C., Whitehead S.N., Dhani S.U., D'Antonio C., Huan L.J., Bennett S.A., Snead O.C., Bear C.E. (2010). Disruption of ClC-2 expression is associated with progressive neurodegeneration in aging mice. Neuroscience.

[17] Nehrke K., Arreola J., Nguyen H.V., Pilato J., Richardson L., Okunade G., Baggs R., Shull G.E., Melvin J.E. (2002). Loss of hyperpolarization-activated Cl^−^ current in salivary acinar cells from *Clcn2* knockout mice. J. Biol. Chem..

[18] Edwards M.M., Marin de Evsikova C., Collin G.B., Gifford E., Wu J., Hicks W.L., Whiting C., Varvel N.H., Maphis N., Lamb B.T., Naggert J.K., Nishina P.M., Peachey N.S. (2010). Photoreceptor degeneration, azoospermia, leukoencephalopathy, and abnormal RPE cell function in mice expressing an early stop mutation in *CLCN2*. Invest. Ophthalmol. Vis. Sci..

[19] Depienne C., Bugiani M., Dupuits C., Galanaud D., Touitou V., Postma N., van Berkel C., Polder E., Tollard E., Darios F., Brice A., de Die-Smulders C.E., Vles J.S., Vanderver A., Uziel G. (2013). Brain white matter oedema due to ClC-2 chloride channel deficiency: an observational analytical study. Lancet Neurol..

[20] Giorgio E., Vaula G., Benna P., Lo Buono N., Eandi C.M., Dino D., Mancini C., Cavalieri S., Di Gregorio E., Pozzi E., Ferrero M., Giordana M.T., Depienne C., Brusco A. (2017). A novel homozygous change of *CLCN2* (p.His590Pro) is associated with a subclinical form of leukoencephalopathy with ataxia (LKPAT). J. Neurol. Neurosurg. Psychiatry.

[21] Hanagasi H.A., Bilgic B., Abbink T.E., Hanagasi F., Tufekcioglu Z., Gurvit H., Basak N., van der Knaap M.S., Emre M. (2015). Secondary paroxysmal kinesigenic dyskinesia associated with *CLCN2* gene mutation. Parkinsonism Relat. Disord..

[22] Guo Z., Lu T., Peng L., Cheng H., Peng F., Li J., Lu Z., Chen S., Qiu W. (2019). *CLCN2*-related leukoencephalopathy: a case report and review of the literature. BMC Neurol..

[23] Di Bella D., Pareyson D., Savoiardo M., Farina L., Ciano C., Caldarazzo S., Sagnelli A., Bonato S., Nava S., Bresolin N., Tedeschi G., Taroni F., Salsano E. (2014). Subclinical leukodystrophy and infertility in a man with a novel homozygous *CLCN2* mutation. Neurology.

[24] López-Hernández T., Ridder M.C., Montolio M., Capdevila-Nortes X., Polder E., Sirisi S., Duarri A., Schulte U., Fakler B., Nunes V., Scheper G.C., Martínez A., Estévez R., van der Knaap M.S. (2011). Mutant GlialCAM causes megalencephalic leukoencephalopathy with subcortical cysts, benign familial macrocephaly, and macrocephaly with retardation and autism. Am. J. Hum. Genet..

[25] Leegwater P.A., Yuan B.Q., van der Steen J., Mulders J., Konst A.A., Boor P.K., Mejaski-Bosnjak V., van der Maarel S.M., Frants R.R., Oudejans C.B., Schutgens R.B., Pronk J.C., van der Knaap M.S. (2001). Mutations of *MLC1* (KIAA0027), encoding a putative membrane protein, cause megalencephalic leukoencephalopathy with subcortical cysts. Am. J. Hum. Genet..

[26] Scholl U.I., Stolting G., Schewe J., Thiel A., Tan H., Nelson-Williams C., Vichot A.A., Jin S.C., Loring E., Untiet V., Yoo T., Choi J., Xu S., Wu A., Kirchner M. (2018). *CLCN2* chloride channel mutations in familial hyperaldosteronism type II. Nat. Genet..

[27] Fernandes-Rosa F.L., Daniil G., Orozco I.J., Göppner C., El Zein R., Jain V., Boulkroun S., Jeunemaitre X., Amar L., Lefebvre H., Schwarzmayr T., Strom T.M., Jentsch T.J., Zennaro M.C. (2018). A gain-of-function mutation in the *CLCN2* chloride channel gene causes primary aldosteronism. Nat. Genet..

[28] Göppner C., Orozco I.J., Hoegg-Beiler M.B., Soria A.H., Hübner C.A., Fernandes-Rosa F.L., Boulkroun S., Zennaro M.C., Jentsch T.J. (2019). Pathogenesis of hypertension in a mouse model for human *CLCN2* related hyperaldosteronism. Nat. Commun..

[29] Schwenk F., Baron U., Rajewsky K. (1995). A cre-transgenic mouse strain for the ubiquitous deletion of *loxP*-flanked gene segments including deletion in germ cells. Nucleic Acids Res..

[30] Lécureuil C., Fontaine I., Crepieux P., Guillou F. (2002). Sertoli and granulosa cell-specific Cre recombinase activity in transgenic mice. Genesis.

[31] Sadate-Ngatchou P.I., Payne C.J., Dearth A.T., Braun R.E. (2008). Cre recombinase activity specific to postnatal, premeiotic male germ cells in transgenic mice. Genesis.

[32] Mori M., Metzger D., Garnier J.M., Chambon P., Mark M. (2002). Site-specific somatic mutagenesis in the retinal pigment epithelium. Invest. Ophthalmol. Vis. Sci..

[33] Thanos A., Morizane Y., Murakami Y., Giani A., Mantopoulos D., Kayama M., Roh M.I., Michaud N., Pawlyk B., Sandberg M., Young L.H., Miller J.W., Vavvas D.G. (2012). Evidence for baseline retinal pigment epithelium pathology in the *Trp1*-Cre mouse. Am. J. Pathol..

[34] Földy C., Lee S.H., Morgan R.J., Soltesz I. (2010). Regulation of fast-spiking basket cell synapses by the chloride channel ClC-2. Nat. Neurosci..

[35] Rinke I., Artmann J., Stein V. (2010). ClC-2 voltage-gated channels constitute part of the background conductance and assist chloride extrusion. J. Neurosci..

[36] Sik A., Smith R.L., Freund T.F. (2000). Distribution of chloride channel-2-immunoreactive neuronal and astrocytic processes in the hippocampus. Neuroscience.

[37] Makara J.K., Rappert A., Matthias K., Steinhäuser C., Spät A., Kettenmann H. (2003). Astrocytes from mouse brain slices express ClC-2-mediated Cl^−^ currents regulated during development and after injury. Mol. Cell Neurosci..

[38] Lappe-Siefke C., Goebbels S., Gravel M., Nicksch E., Lee J., Braun P.E., Griffiths I.R., Nave K.A. (2003). Disruption of *Cnp1* uncouples oligodendroglial functions in axonal support and myelination. Nat. Genet..

[39] Tao J., Wu H., Lin Q., Wei W., Lu X.H., Cantle J.P., Ao Y., Olsen R.W., Yang X.W., Mody I., Sofroniew M.V., Sun Y.E. (2011). Deletion of astroglial Dicer causes non-cell-autonomous neuronal dysfunction and degeneration. J. Neurosci..

[40] Mori T., Tanaka K., Buffo A., Wurst W., Kühn R., Götz M. (2006). Inducible gene deletion in astroglia and radial glia--a valuable tool for functional and lineage analysis. Glia.

[41] Winchenbach J., Duking T., Berghoff S.A., Stumpf S.K., Hulsmann S., Nave K.A., Saher G. (2016). Inducible targeting of CNS astrocytes in *Aldh1l1*-CreERT2 BAC transgenic mice. F1000Res.

[42] Capdevila-Nortes X., Jeworutzki E., Elorza-Vidal X., Barrallo-Gimeno A., Pusch M., Estévez R. (2015). Structural determinants of interaction, trafficking and function in the ClC-2/MLC1 subunit GlialCAM involved in leukodystrophy. J. Physiol..

[43] Lundgaard I., Osorio M.J., Kress B.T., Sanggaard S., Nedergaard M. (2014). White matter astrocytes in health and disease. Neuroscience.

[44] Orthmann-Murphy J.L., Abrams C.K., Scherer S.S. (2008). Gap junctions couple astrocytes and oligodendrocytes. J. Mol. Neurosci..

[45] Boussouar F., Benahmed M. (2004). Lactate and energy metabolism in male germ cells. Trends Endocrinol. Metab..

[46] Bernardino R.L., D'Souza W.N., Rato L., Rothstein J.L., Dias T.R., Chui D., Wannberg S., Alves M.G., Oliveira P.F. (2019). Knockout of MCT1 results in total absence of spermatozoa, sex hormones dysregulation, and morphological alterations in the testicular tissue. Cell Tissue Res..

[47] Jutte N.H., Jansen R., Grootegoed J.A., Rommerts F.F., Clausen O.P., van der Molen H.J. (1982). Regulation of survival of rat pachytene spermatocytes by lactate supply from Sertoli cells. J. Reprod. Fertil..

[48] França L.R., Hess R.A., Dufour J.M., Hofmann M.C., Griswold M.D. (2016). The Sertoli cell: one hundred fifty years of beauty and plasticity. Andrology.

[49] Griswold M.D. (2018). 50 years of spermatogenesis: Sertoli cells and their interactions with germ cells. Biol. Reprod..

[50] Mital P., Hinton B.T., Dufour J.M. (2011). The blood-testis and blood-epididymis barriers are more than just their tight junctions. Biol. Reprod..

[51] Caceres P.S., Rodriguez-Boulan E. (2020). Retinal pigment epithelium polarity in health and blinding diseases. Curr. Opin. Cell Biol..

[52] Lakkaraju A., Umapathy A., Tan L.X., Daniele L., Philp N.J., Boesze-Battaglia K., Williams D.S. (2020). The cell biology of the retinal pigment epithelium. Prog. Retin. Eye Res..

[53] Penberthy K.K., Lysiak J.J., Ravichandran K.S. (2018). Rethinking phagocytes: clues from the retina and testes. Trends Cell Biol..

[54] Felmlee M.A., Jones R.S., Rodriguez-Cruz V., Follman K.E., Morris M.E. (2020). Monocarboxylate transporters (SLC16): function, regulation, and role in health and disease. Pharmacol. Rev..

[55] De Jesús-Pérez J.J., Castro-Chong A., Shieh R.C., Hernández-Carballo C.Y., De Santiago-Castillo J.A., Arreola J. (2016). Gating the glutamate gate of CLC-2 chloride channel by pore occupancy. J. Gen. Physiol..

[56] Niemeyer M.I., Cid L.P., Zuñiga L., Catalán M., Sepúlveda F.V. (2003). A conserved pore-lining glutamate as a voltage- and chloride-dependent gate in the ClC-2 chloride channel. J. Physiol..

[57] Catalán M., Niemeyer M.I., Cid L.P., Sepúlveda F.V. (2004). Basolateral ClC-2 chloride channels in surface colon epithelium: regulation by a direct effect of intracellular chloride. Gastroenterology.

[58] Lück J.C., Puchkov D., Ullrich F., Jentsch T.J. (2018). LRRC8/VRAC anion channels are required for late stages of spermatid development in mice. J. Biol. Chem..

[59] Planells-Cases R., Lutter D., Guyader C., Gerhards N.M., Ullrich F., Elger D.A., Kucukosmanoglu A., Xu G., Voss F.K., Reincke S.M., Stauber T., Blomen V.A., Vis D.J., Wessels L.F., Brummelkamp T.R. (2015). Subunit composition of VRAC channels determines substrate specificity and cellular resistance to Pt-based anti-cancer drugs. EMBO J..

[60] Zhou C., Chen X., Planells-Cases R., Chu J., Wang L., Cao L., Li Z., Lopez-Cayuqueo K.I., Xie Y., Ye S., Wang X., Ullrich F., Ma S., Fang Y., Zhang X. (2020). Transfer of cGAMP into bystander cells via LRRC8 volume-regulated anion channels augments STING-mediated interferon responses and anti-viral immunity. Immunity.

[61] Nilius B., Eggermont J., Voets T., Buyse G., Manolopoulos V., Droogmans G. (1997). Properties of volume-regulated anion channels in mammalian cells. Prog. Biophys. Mol. Biol..

[62] Nilius B., Prenen J., Droogmans G. (1998). Modulation of volume-regulated anion channels by extra- and intracellular pH. Pflügers Arch..

[63] Lutter D., Ullrich F., Lueck J.C., Kempa S., Jentsch T.J. (2017). Selective transport of neurotransmitters and modulators by distinct volume-regulated LRRC8 anion channels. J. Cell Sci..

[64] Bao J., Perez C.J., Kim J., Zhang H., Murphy C.J., Hamidi T., Jaubert J., Platt C.D., Chou J., Deng M., Zhou M.H., Huang Y., Gaitán-Peñas H., Guénet J.L., Lin K. (2018). Deficient LRRC8A-dependent volume-regulated anion channel activity is associated with male infertility in mice. JCI insight.

[65] Zhang Y., Kittredge A., Ward N., Ji C., Chen S., Yang T. (2018). ATP activates bestrophin ion channels through direct interaction. Nat. Commun..

[66] Johnson A.A., Guziewicz K.E., Lee C.J., Kalathur R.C., Pulido J.S., Marmorstein L.Y., Marmorstein A.D. (2017). Bestrophin 1 and retinal disease. Prog. Retin. Eye Res..

[67] Marmorstein L.Y., Wu J., McLaughlin P., Yocom J., Karl M.O., Neussert R., Wimmers S., Stanton J.B., Gregg R.G., Strauss O., Peachey N.S., Marmorstein A.D. (2006). The light peak of the electroretinogram is dependent on voltage-gated calcium channels and antagonized by bestrophin (best-1). J. Gen. Physiol..

[68] Xiao Q., Hartzell H.C., Yu K. (2010). Bestrophins and retinopathies. Pflugers Arch..

[69] Catalán M., Cornejo I., Figueroa C.D., Niemeyer M.I., Sepúlveda F.V., Cid L.P. (2002). ClC-2 in Guinea pig colon: mRNA, immunolabeling, and functional evidence for surface epithelium localization. Am. J. Physiol. Gastrointest. Liver Physiol..

[70] Zdebik A.A., Cuffe J., Bertog M., Korbmacher C., Jentsch T.J. (2004). Additional disruption of the ClC-2 Cl^−^ channel does not exacerbate the cystic fibrosis phenotype of CFTR mouse models. J. Biol. Chem..

[71] Butt A.M., Kalsi A. (2006). Inwardly rectifying potassium channels (Kir) in central nervous system glia: a special role for Kir4.1 in glial functions. J. Cell. Mol. Med..

[72] Neusch C., Rozengurt N., Jacobs R.E., Lester H.A., Kofuji P. (2001). Kir4.1 potassium channel subunit is crucial for oligodendrocyte development and *in vivo* myelination. J. Neurosci..

[73] Nagelhus E.A., Mathiisen T.M., Ottersen O.P. (2004). Aquaporin-4 in the central nervous system: cellular and subcellular distribution and coexpression with KIR4.1. Neuroscience.

[74] Schirmer L., Mobius W., Zhao C., Cruz-Herranz A., Ben Haim L., Cordano C., Shiow L.R., Kelley K.W., Sadowski B., Timmons G., Probstel A.K., Wright J.N., Sin J.H., Devereux M., Morrison D.E. (2018). Oligodendrocyte-encoded Kir4.1 function is required for axonal integrity. eLife.

[75] Menichella D.M., Majdan M., Awatramani R., Goodenough D.A., Sirkowski E., Scherer S.S., Paul D.L. (2006). Genetic and physiological evidence that oligodendrocyte gap junctions contribute to spatial buffering of potassium released during neuronal activity. J. Neurosci..

[76] Neusch C., Papadopoulos N., Muller M., Maletzki I., Winter S.M., Hirrlinger J., Handschuh M., Bahr M., Richter D.W., Kirchhoff F., Hulsmann S. (2006). Lack of the Kir4.1 channel subunit abolishes K^+^ buffering properties of astrocytes in the ventral respiratory group: impact on extracellular K^+^ regulation. J. Neurophysiol..

[77] Menichella D.M., Goodenough D.A., Sirkowski E., Scherer S.S., Paul D.L. (2003). Connexins are critical for normal myelination in the CNS. J. Neurosci..

[78] Walz W. (2002). Chloride/anion channels in glial cell membranes. Glia.

[79] Haj-Yasein N.N., Jensen V., Vindedal G.F., Gundersen G.A., Klungland A., Ottersen O.P., Hvalby O., Nagelhus E.A. (2011). Evidence that compromised K_+_ spatial buffering contributes to the epileptogenic effect of mutations in the human Kir4.1 gene (*KCNJ10*). Glia.

[80] Leegwater P.A., Boor P.K., Yuan B.Q., van der Steen J., Visser A., Konst A.A., Oudejans C.B., Schutgens R.B., Pronk J.C., van der Knaap M.S. (2002). Identification of novel mutations in MLC1 responsible for megalencephalic leukoencephalopathy with subcortical cysts. Hum. Genet..

[81] Catalán M.A., Flores C.A., González-Begne M., Zhang Y., Sepúlveda F.V., Melvin J.E. (2012). Severe defects in absorptive ion transport in distal colons of mice that lack ClC-2 channels. Gastroenterology.

[82] Mortimer D., Curtis E.F., Miller R.G. (1987). Specific labelling by peanut agglutinin of the outer acrosomal membrane of the human spermatozoon. J. Reprod. Fertil..

