# Supplementary material for: Cellular basis of ClC-2 Cl− channel–related brain and testis pathologies
Source: J Biol Chem. 2020 Nov 23;296:100074. doi: 10.1074/jbc.RA120.016031 (PMC7949093; doi:10.1074/jbc.RA120.016031)
Supplement: Supplementary Figures and Tables [file mmc1.pdf]

Supporting Information for

‘Cellular basis of ClC-2 Cl⁻ channel-related brain and testis pathologies’

by Göppner, Soria, Hoegg-Beiler & Jentsch

Supplementary Figures

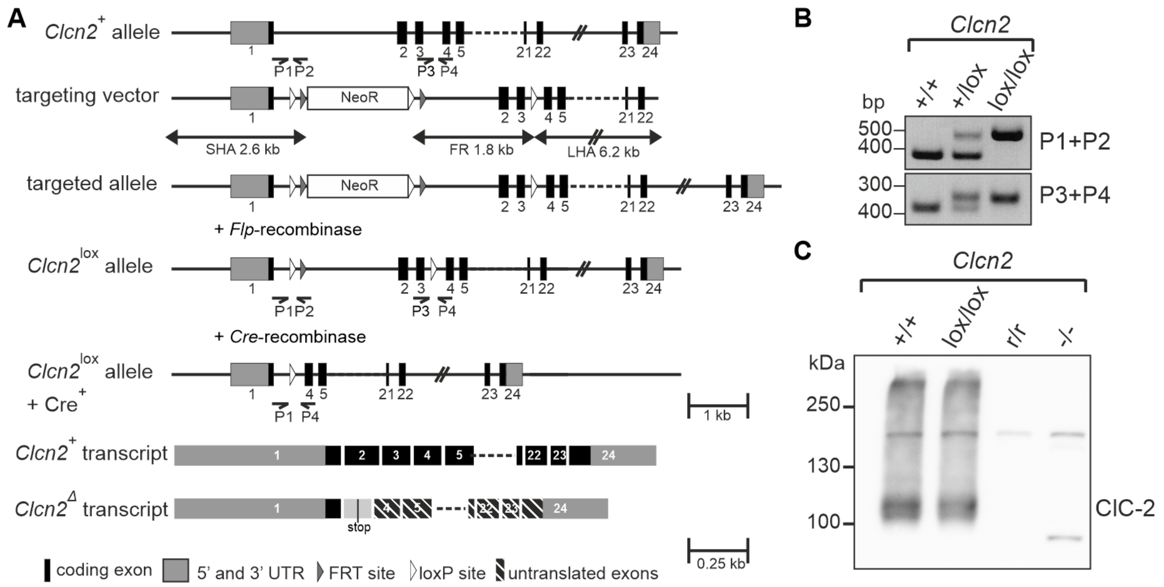

Figure S1: Generation of the conditional *Clcn2* knockout mouse model.

A To generate a conditional *Clcn2* knock-out mouse model (*Clcn2*$^{lox/lox}$) a neomycin resistance cassette (NeoR) for ES cell selection flanked by FRT sites and loxP sites flanking exons 2 and 3 were inserted into a plasmid containing the *Clcn2* gene. The NeoR cassette was removed by *Flpe* recombination resulting in *Clcn2*^{lox} allele. Crossing of the conditional *Clcn2* knock-out mice to mice expressing the Cre-recombinase in a cell-type specific manner leads to removal of exons 2 and 3 in the respective cell type. This results in a frame shift and early stop codon leading to abortive protein production. ***B*** PCR genotyping of the mice screening for the two loxP sites using two primer pairs (P1 and P2; P3 and P4) as indicated in panel a. ***C*** Western blot analysis of ClC-2 of membrane fractions isolated from brain tissue of adult *Clcn2*$^{+/+}$, *Clcn2*$^{lox/lox}$ mice and *Clcn2*$^{lox/lox}$ mice expressing a Cre-recombinase in all cells (*Clcn2*$^{r/r}$) and constitutive *Clcn2* knock-out mice (*Clcn2*$^{-/-}$). Equal amounts of protein were loaded as determined by BCA-assay. This blot is representative for two independent experiments.

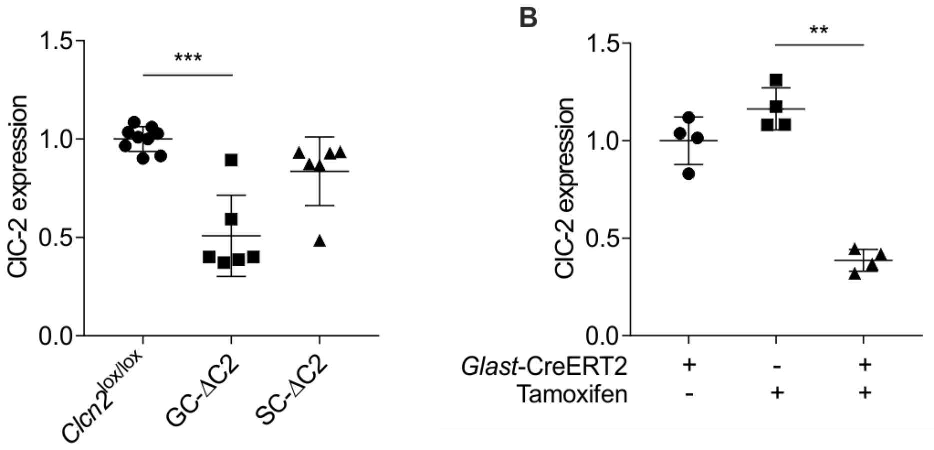

Figure S2: Quantification of Western blots corresponding to main figures 1*A* and 3*B*.

Densitometric quantification of ClC-2 protein bands in Fig. 1*A* (***A***) and Fig. 3*B* (***B***). Data presented as mean ± SD, normalized to the mean values of *Clcn2*$^{lox/lox}$ (*A*) or non-induced *Glast*-Cre^{ERT2}; *Clcn2*$^{lox/lox}$ (*B*). Kruskal-Wallis test with Dunn's multiple comparison test, adjusted p values: $**p=0.01$, $***p < 0.001$.

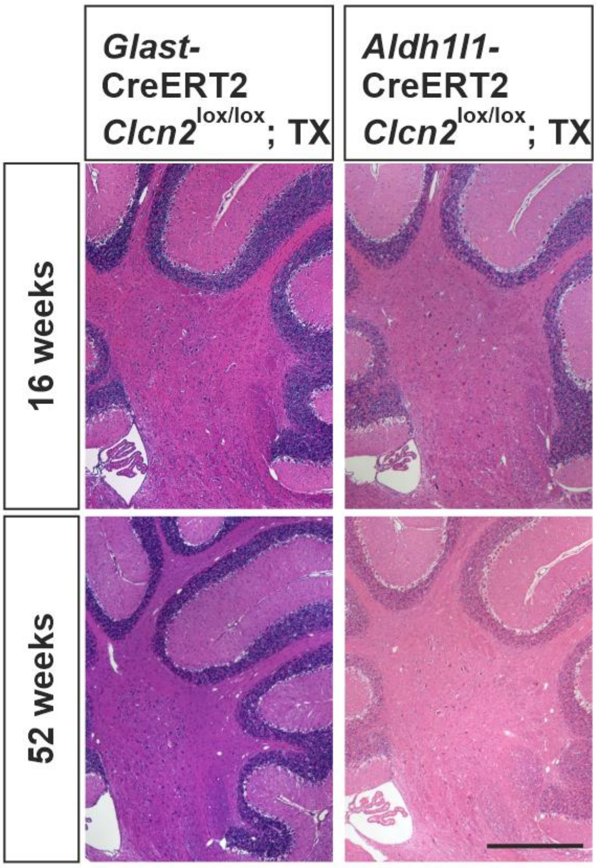

Figure S3: No myelin vacuolization in inducible astrocyte-specific *Clcn2* knock-out mouse models.

H&E stained sagittal brain sections of the myelin layer of the cerebellum of astrocyte-specific ClC-2 knock-out mice (*Glast-CreERT2; Clcn2*$^{lox/lox}$ induced with tamoxifen (TX), *Aldh1l1-CreERT2*; *Clcn2*$^{lox/lox}$ TX) in the age of 16 and 52 weeks. n = 2 – 4. Scale bar: 500 µm.

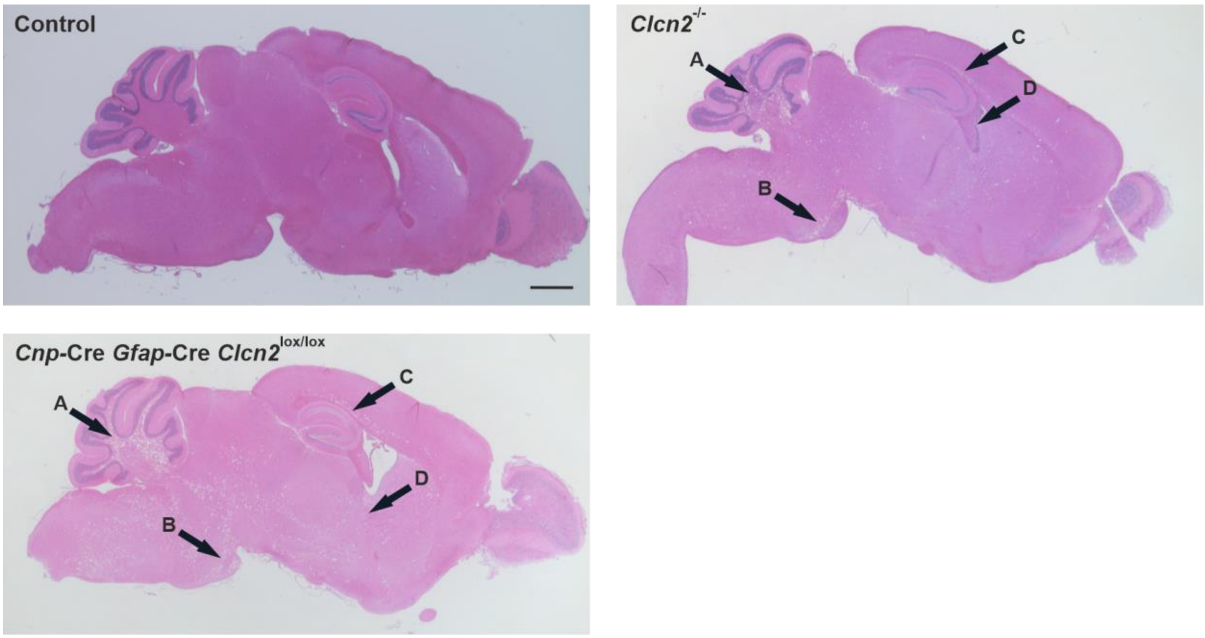

Figure S4: Overview of myelin vacuolization phenotype of oligodendrocyte/astrocyte-specific *Clcn2* knock-out mice.

H&E stained sagittal whole brain sections of 52-week-old control, *Clcn2*$^{-/-}$ and oligodendrocyte-/astrocyte-specific ClC-2 knock-out (*Cnp-Cre;Gfap-Cre;Clcn2*$^{lox/lox}$) mice. Arrows indicate myelin vacuolization in cerebellum (A), brain stem (B), corpus callosum (C) and in fiber tracts (D). n = 3 – 4. Scale bars: 1 mm.

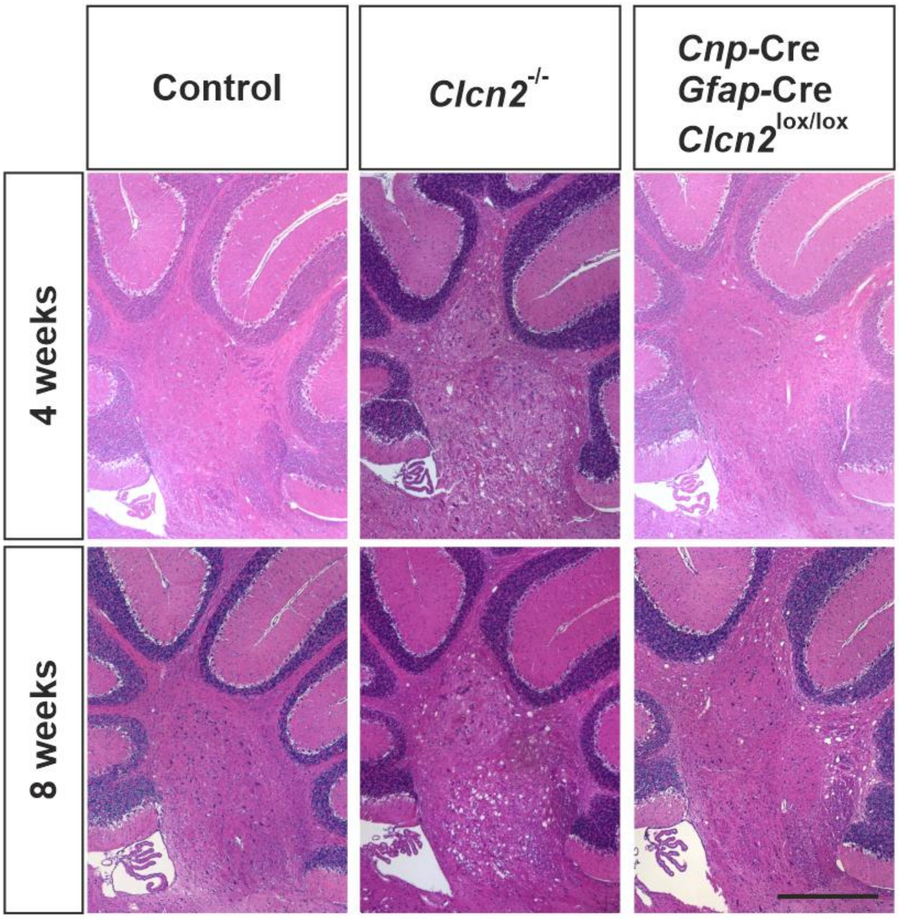

Figure S5: Myelin vacuolization in young oligodendrocyte/astrocyte-specific *Clcn2* knock-out compared to constitutive *Clcn2* knock-out mice.

H&E stained sagittal brain sections of the myelin layer of the cerebellum of control, *Clcn2*$^{-/-}$ and oligodendrocyte-/astrocyte-specific ClC-2 knockout (*Cnp-Cre;Gfap-Cre;Clcn2*$^{lox/lox}$) mice at the age of four and eight weeks. n = 2 – 3. Scale bar: 500 µm.

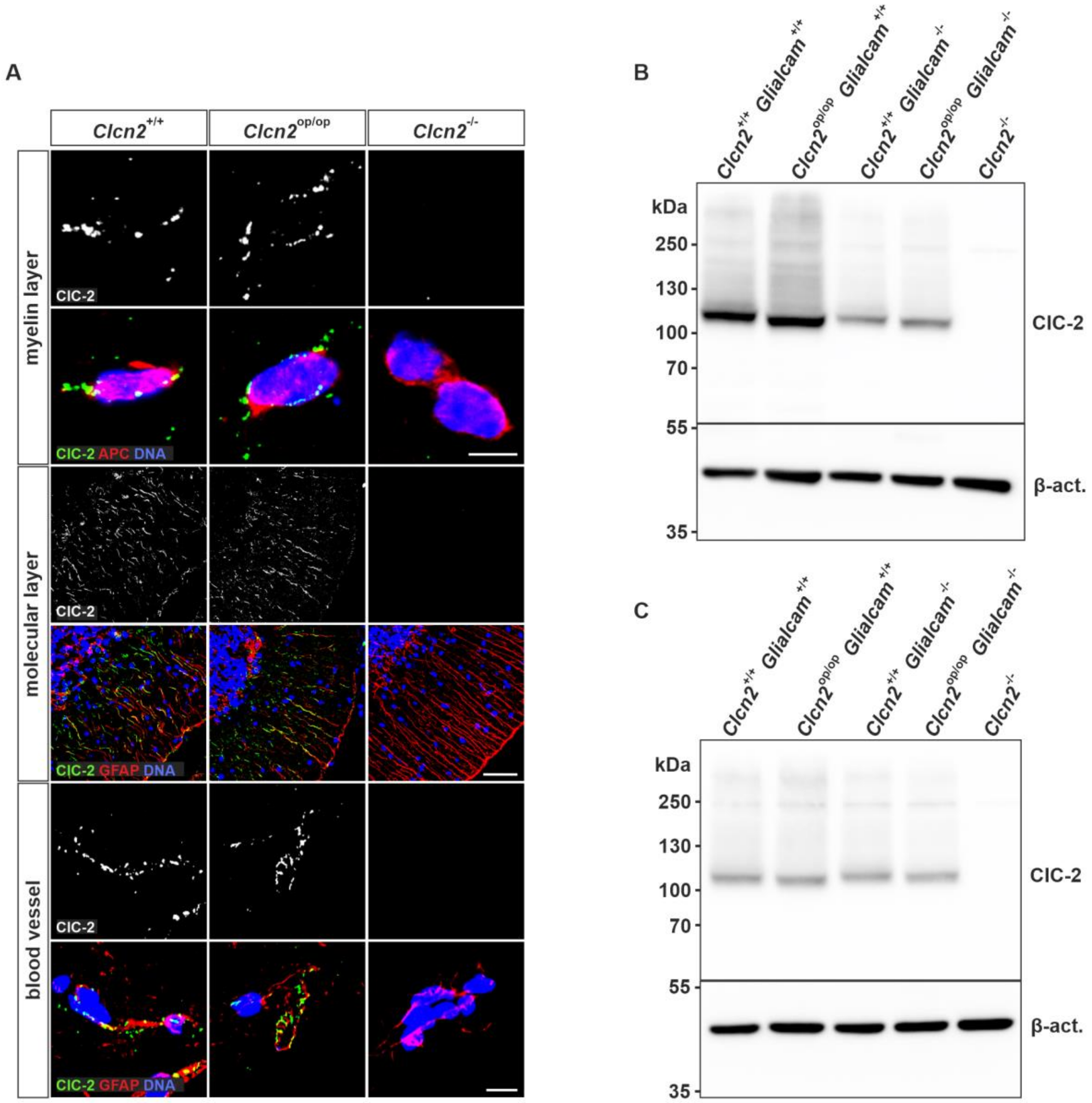

Figure S6: Unchanged ClC-2 expression and localization in the brain of *Clcn2*$^{op/op}$ mice.

A Immunofluorescent labeling of ClC-2 (green) in brain cryosections of adult wildtype, *Clcn2*$^{+/op}$, *Clcn2*$^{op/op}$ and *Clcn2*$^{-/-}$ mice. Co-staining for adenomatous polyposis coli (APC, red) visualizes the cell bodies of oligodendrocytes in the myelin layer of the cerebellum. Co-staining for the astrocytic cytoskeletal protein GFAP (red) visualizes Bergmann glia on the molecular layer of the cerebellum as well as astrocytic endfeet at blood vessels next to the hippocampus. DNA in the nuclei was stained with DAPI (blue). Scale bars: 5 µm (staining in myelin layer), 50 µm (staining in molecular layer), 10 µm (staining of blood vessels). ***B* and *C*** Western blot analysis of ClC-2 of membrane fractions isolated from cerebellum (*B*) and brain without cerebellum (*C*) of adult wildtype (*Clcn2*$^{+/+}$;*Glialcam*$^{+/+}$), *Clcn2*$^{op/op}$;*Glialcam*$^{+/+}$, *Clcn2*$^{+/+}$; *Glialcam*$^{-/-}$and *Clcn2*$^{op/op}$;*Glialcam*$^{-/-}$ mice, with protein lysate from *Clcn2*$^{-/-}$ mice as control. Equal amounts of protein, as determined by BCA-assay, were loaded with β-actin serving as loading control. This blot is representative for two independent experiments.
